# Monetary Diet Cost, Diet Quality, and Parental Socioeconomic Status in Spanish Youth

**DOI:** 10.1371/journal.pone.0161422

**Published:** 2016-09-13

**Authors:** Helmut Schröder, Santiago F. Gomez, Lourdes Ribas-Barba, Carmen Pérez-Rodrigo, Rowaedh Ahmed Bawaked, Montserrat Fíto, Lluis Serra-Majem

**Affiliations:** 1 Cardiovascular Risk and Nutrition Research Group (CARIN), IMIM (Hospital del Mar Medical Research Institute), Barcelona, Spain; 2 CIBER Epidemiology and Public Health (CIBERESP), Instituto de Salud Carlos III, Madrid, Spain; 3 AQuAS / ASPCAT, Departament de Salut, Generalitat de Catalunya, Barcelona, Spain; 4 PhD Programme in Biomedicine, Universidad Pompeu Fabra, Barcelona, Spain; 5 Fundación para la Investigación Nutricional (Nutrition Research Foundation), Barcelona, Spain; 6 CIBER Physiopathology of Obesity and Nutrition (CIBEROBN), Instituto de Salud Carlos III, Madrid, Spain; 7 FIDEC Foundation, University of the Basque Country, Bilbao, Spain; 8 Reseach Institute of Biomedical and Health Sciences, University of Las Palmas de Gran Canaria, Las Palmas de Gran Canaria, Spain; Georgia Regents University, UNITED STATES

## Abstract

**Background:**

Using a food-based analysis, healthy dietary patterns in adults are more expensive than less healthy ones; studies are needed in youth. Therefore, the objective of the present study was to determine relationships between monetary daily diet cost, diet quality, and parental socioeconomic status.

**Design and Methods:**

Data were obtained from a representative national sample of 3534 children and young people in Spain, aged 2 to 24 years. Dietary assessment was performed with a 24-hour recall. Mediterranean diet adherence was measured by the KIDMED questionnaire. Average food cost was calculated from official Spanish government data. Monetary daily diet cost was expressed as euros per day (€/d) and euros per day standardized to a 1000kcal diet (€/1000kcal/d).

**Results:**

Mean monetary daily diet cost was 3.16±1.57€/d (1.56±0.72€/1000kcal/d). Socioeconomic status was positively associated with monetary daily diet cost and diet quality measured by the KIDMED index (€/d and €/1000kcal/d, *p*<0.019). High Mediterranean diet adherence (KIDMED score 8–12) was 0.71 €/d (0.28€/1000kcal/d) more expensive than low compliance (KIDMED score 0–3). Analysis for nonlinear association between the KIDMED index and monetary daily diet cost per1000kcal showed no further cost increases beyond a KIDMED score of 8 (linear *p*<0.001; nonlinear *p* = 0.010).

**Conclusion:**

Higher monetary daily diet cost is associated with healthy eating in Spanish youth. Higher socioeconomic status is a determinant for higher monetary daily diet cost and quality.

## Introduction

A healthy diet is paramount for physical and mental health [1,2]. The Mediterranean diet is an excellent example of a healthy diet: it provides adequate food and nutrient intakes, high nutrient density, and reduces the risk of non-communicable diseases such as diabetes, cancer, and cardiovascular disease in adults [3, 4]. Moreover, high adherence to this healthy dietary pattern has been associated with better cardiovascular health in children and young adults [5–7]. Therefore, adaption to this healthy eating pattern at young ages lays the foundation for a healthy lifestyle. Furthermore, identification of determinants that may limit adherence to a healthy diet is an important consideration.

Adoption of a specific diet is mainly determined by taste, convenience, and price [8], and higher adherence to the Mediterranean diet has been associated with higher dietary costs in Spanish adults [9]; other healthy dietary patterns also are more expensive than unhealthy choices [10]. This partially explains why low diet quality is more often found in segments of the population with the lowest socioeconomic status (SES). Less is known about the association of monetary diet cost and healthy eating in children and youth, and about the relationship to parental SES [11, 12]. The KIDMED index was developed to measure adherence to the Mediterranean diet in children and youth, based on the principles that sustain Mediterranean dietary patterns and those that undermine it [13], and has been used in various young populations [14–17].

Based on previous findings in adults, we hypothesized that adherence to the Mediterranean diet is positively associated with monetary daily diet cost in Spanish youth. Therefore, we determined the association between the monetary cost of the diet, both in euros per day (€/d) and euros per 1000 kcal per day (€/1000kcal/d), and total diet quality as measured by adherence to the Mediterranean diet, in a nationwide representative sample of Spanish children, adolescents, and young adults. In addition, we analyzed the relationship between parental SES and monetary daily diet cost (€/d and €/1000kcal/d).

## Methods

### Study population

The enKid study on nutritional status and food habits of Spanish children and young people, carried out between 1998 and 2000, was a cross-sectional survey of the Spanish population aged 2 to 24 years (n = 3534, 1629 boys and 1905 girls), selected by multistage random sampling procedures based on an official population census. The theoretical sample size was set at 5500 individuals, taking into account an anticipated 70% participation rate, which would result in a sample of approximately 3850 individuals. The study protocol was approved by the ethics committee of the Spanish Society of Community Nutrition. Written informed consent was obtained for each participant (parental consent for those younger than 18 years).

### Food price database

Food prices were obtained from the official food price database of the Spanish Ministry of the Economy and Competitiveness [18]. The average prices for many food items (not including commercial fast foods) are updated every month in this database. For this study, we chose the average accumulated prices reported in 2000. Food price data were obtained from Spanish municipalities, selected according to demographic criteria to ensure a representative sample. The municipalities include the 23 provincial capitals (Alicante, Almeria, Badajoz, Barcelona, Bilbao, Cordova, Granada, Las Palmas of Grand Canary, León, Madrid, Malaga, Murcia, Oviedo, Palma, Pamplona, Salamanca, San Sebastian, Santander, Seville, Santa Cruz de Tenerife, Valencia, Valladolid, Saragossa) and the cities of Vigo, Santiago, Gijon and Sherry. The most representative foods of each food group were selected by an expert panel for inclusion in the database, taking into account criteria such as products that are readily and permanently available, for which prices are easily observable, and that are usually consumed by the population. The final database includes 274 foods and beverages, with prices expressed as price (€) per kg unless otherwise indicated.

### Monetary diet cost calculation

Dietary intake information based on a single 24-hour recall was collected at each participant’s home, using household measures to estimate portion sizes [19]. The mother or other person responsible for feeding the children helped participants younger than 8 years to complete the recall. Additional information on food descriptions and portion sizes was gathered as required on site. Day of collection was randomly assigned, including weekends and non-school days.

Monetary cost of the diet was calculated by multiplying gram amounts of food consumed by each participant by the corresponding price per gram and adding up the total sum for each participant. In addition, we calculated the monetary daily cost of 6 food groups (cereals, dairy, meat and sausages, fish, fruits and vegetables, and pastry and sweets) as a percentage of total monetary daily diet cost. Total monetary daily diet cost was computed in two price metrics: i) €/d and ii) €/1000kcal/d. The cost-per-day metric is probably the most relevant for consumer foods choice, whereas standardization to a 1000kcal diet facilitates comparison with other studies

### Adherence to the Mediterranean diet

Adherence to the Mediterranean diet was estimated by the KIDMED index, created to estimate adherence to the Mediterranean diet in children and young adults. The KIDMED index was derived from a 16-item questionnaire administered separately from the 24-hour recalls as part of the EnKid survey [13]. Items denoting lower adherence were assigned a value of -1 (4 items) and those related to higher adherence were scored +1 (12 items). Scores range from -4 to 12, with higher scores indicating greater adherence to the Mediterranean diet [13]. The KIDMED index shows reasonable construct validity [20].

The KIDMED index was based on the principles that sustain Mediterranean dietary patterns and those that undermine it. Items denoting lower adherence were assigned a value of -1 [4 items: Goes more than once a week to a fast-food restaurant; skips breakfast; has commercially baked goods or pastries for breakfast; takes sweets and candy several times every day]. Those related to higher adherence were scored +1 [12 items: Consumes fruit or fruit juice every day; has a second fruit every day; has fresh or cooked vegetables regularly once a day; has fresh or cooked vegetables more than once a day; consumes fish regularly; likes pulses and eats them more than once a week; consumes pasta or rice almost every day (5 or more times per week); has cereals or grains (bread, etc.) for breakfast; consumes nuts regularly (at least 2–3 times per week); uses olive oil at home; has a dairy product for breakfast (yoghurt, milk, etc.); Eats a total of two yoghurts and/or some cheese (40 g) daily]. Scores range from -4 to 12, with higher scores indicating greater adherence to the Mediterranean diet. A low, intermediate, and high adherence to the Mediterranean diet was defined as scoring below 4, between 4 and 7, and more than 7 points for the KIDMED index, respectively.

### Energy misreporting

Basel metabolic rate (BMR) was estimated with Schofield’s equations based on sex, age, weight, and height [21]. Implausible reporters of energy intake were identified by replacing Goldberg’s single cut-off [22] with age- and sex-specific cut-offs for children. The cut-off values are the 95% confidence limits of the agreement between Physical Activity Level (PAL) and the ratio of energy intake to BMR. The following formula was used:

$$\text{Cutoff}=PAL\;x\;exp\left\lceil \pm 1.96\;x\;\frac{\left(\frac{S}{100}\right)}{\sqrt{n}}\right\rceil$$

Where

$$s=\sqrt{\left[\frac{CV_{wEI}^{2}}{d}+CV_{wBMR}^{2}+\;CV_{tP}^{2}\right]}$$

Intra-individual variations of energy intake (CV^2^_wEI_) and BMR (CV^2^_wBMR_) and inter-individual variation in physical activity level (CV^2^_wtP_) were calculated using sex- and age-specific reference values [23–25]. The single Goldberg PAL of 1.55 was replaced by sex- and age-dependent PAL for adolescents. We estimated dietary intake by one 24-hour recall and set the number of days (d) to 1.

### Socioeconomic status

Determination of SES was based on maternal educational level: i) no education (never went to school), ii) primary education not completed, iii) primary education, iv) secondary education, and v) university.

### Statistics

We conducted general linear modeling procedures to compare participant sociodemographic and dietary characteristics by quintiles of monetary diet cost expressed in two price metrics: €/d and €/1000kcal/d. *P* values were obtained by ANOVA and Pearson chi square for normal continuous and categorical variables, respectively.

To determine the association of diet quality and monetary daily diet cost (€/d and €/1000kcal/d), we fitted a general linear model with categories of adherence to the KIDMED index (low, intermediate, and high) as fixed factor and monetary daily diet cost (€/day and €/1000kcal/d) as dependent variable. The model was adjusted for sex, age, region, community size, and energy over- and underreporting. Cubic spline analysis was performed to investigate nonlinear associations between monetary daily diet cost (€/day and €/1000kcal/d) and diet quality measured by the KIDMED index, using the ‘gam’ package in R version 3.0.2.

In a further analysis we were interested in the monetary daily diet cost of selected food groups (€/day and €/1000kcal/d) according to low and high adherence to the KIDMED index. For this purpose, we conducted general linear modeling procedure comparing mean monetary daily diet cost (€/d and €/1000kcal/d) of cereal, vegetable/fruits, meat and sausages, fish, dairy, and pastry and sweets {expressed as percentage of total daily diet cost (€/d and €/1000kcal/d)} according to low and high adherence to the KIDMED index. The model was adjusted for sex, age, region, community size, and energy over- and underreporting. Finally, we conducted multiple linear regression analysis with cubic spline modelling to determine the impact of socioeconomic status measured by maternal education level on monetary daily diet cost (€/day and €/1000kcal/d) and diet quality. Statistical analysis was performed using SPSS version 18∙0. (SPSS Inc. Chicago, Ill., USA). Data are available in S1 Dataset.

## Results

The mean monetary daily diet cost was 3.16±1.57€/d (1.56±0.72€/1000kcal/d), with a higher cost in boys (3.43±1.73€/d) than in girls (2.92±1.37€/d); when standardized to 1000kcal, however, the opposite was true (1.61±0.77€/1000kcal/d in girls vs 1.51±0.66€/1000kcal/d in boys). Monetary daily diet cost (€/d) increased with age (preschoolers, 2.41±1.17€/d; schoolchildren, 2.86±1.26€/d; adolescents, 3.22±1.64€/d; young adults, 3.44±1.62€/d). Energy-adjusted monetary daily diet cost (€/1000 kcal/d) was significantly (p<0.0001) higher in young adults (1.69±0.79€/1000kcal/d) compared to preschoolers (1.49±0.70€/1000kcal/d), schoolchildren (1.44±0.56€/1000 kcal/d), and adolescents (1.46±0.66€/1000kcal/d).

As the monetary daily cost of the diet (€/d) increased, the proportion of girls and the community size decreased and the proportion of high paternal and maternal education increased (Table 1). The Canary Islands showed the lowest proportion of individuals in the highest dietary cost category (Table 1). Similar associations, with the exception of sex and paternal education, were observed for monetary daily diet cost standardized to 1000kcal.

**Table 1:** Demographic and lifestyle characteristics of participants across quintiles of baseline daily monetary diet cost (€/day and €/1000 kcal/day)^1^^,^^2^.

	1^st^ quintile	2^nd^ quintile	3^rd^ quintile	4^th^ quintile	5^th^ quintile	*P*
	^3^(n = 652)	(n = 652)	(n = 654)	(n = 652)	(n = 652)	
	^4^(n = 653)	(n = 652)	(n = 654)	(n = 652)	(n = 651)	
Diet cost (€/day)	1.46 (1.40;1.51)	2.25 (2.19;2.30)	2.87 (2.82;2.92)	3.67 (3.61;3.72)	5.57 (5.52;5.63)	
Diet cost (€/1000kcal/day)	0.83 (0.80;0.86)	1.17 (1.14;1.20)	1.42 (1.39;1.45)	1.74 (1.72;1.77)	2.66 (2.63;2.67)	
Girls (%)						
€/day	62.9 (410)	59.2 (386)	56.7 (370)	47.7 (311)	44.3 (276)	<0.001
€/1000kcal/day	52.8 (342)	51.4 (386)	50.1 (370)	54.2 (311)	60.7 (276)	0.002
Age (y)						
€/day	13.2 (12.8;13.8)	14.2 (13.8;14.7)	15.4 (14.9;15.9)	16.4 (15.9;16.9)	17.5 (17.0;17.9)	<0.001
€/1000kcal/day	14.1 (13.6;14.6)	14.7 (14.2;15.2)	15.5 (15.0;16.0)	15.8 (15.3;16.3)	16.7 (16.3;17.2)	<0.001
Maternal education^5^ %						
€/day	15.4 (99)	17.7 (114)	21.1 (137)	17.5 (113)	21.3 (137)	0.017
€/1000kcal/day	16.7 (108)	16.8 (108)	19.0 (123)	18.9 (122)	21.8 (139)	0.015
	25.4 (162)	23.7 (151)	21.0 (174)	25.9 (166)	27.6 (176)	0.225
Region of Spain (%)^6^						
€/day—Center	21.5 (169)	19.4 (153)	19.4 (153)	18.4 (145)	21.2 (167)	
- Northeast	18.2 (145)	17.9 (143)	19.8 (158)	23.6 (188)	20.5 (163)	
- North	20.9 (151)	22.4 (162)	20.3 (147)	19.1 (138)	17.4 (128)	
- South	16.0 (74)	19.5 (90)	23.4 (108)	19.0 (88)	22.1 (102)	
- East	23.1 (90)	19.0 (74)	16.4 (64)	19.7 (77)	21.8 (85)	
- Canary Islands	22.8 (23)	29.7 (30)	22.8 (23)	15.8 (16)	8.9 (8)	0.003
€/1000kca/day—Center	20.6 (162)	20.2 (159)	18.8 (148)	19.4 (153)	21.0 (165)	
- Northeast	17.9 (143)	18.2 (145)	19.2 (153)	22.8 (182)	21.8 (174)	
- North	23.8 (172)	23.9 (173)	20.6 (149)	16.3 (118)	15.5 (112)	
- South	19.7 (91)	19.5 (90)	18.6 (86)	20.3 (94)	21.9 (101)	
- East	15.9 (62)	17.2 (67)	23.3 (91)	21.5 (84)	22.1 (86)	
- Canary Islands	22.8 (23)	17.8 (18)	25.7 (26)	20.8 (21)	12.9 (13)	0.001
Community size (%)^7^						
€/day <10000	22.8 (164)	21.2 (152)	18.5 (133)	18.4 (132)	19.2 (138)	
10000–49999	23.3 (197)	18.2 (154)	22.5 (190)	17.9 (151)	18.1 (153)	
50000–350000	17.9 (156)	21.3 (186)	20.3 (177)	21.5 (188)	19.0 (166)	
>350000	16.4 (135)	19.4 (160)	18.6 (153)	22.0 (181)	23.7 (195)	0.001
€/1000kcal/day <10000	21.0 (151)	23.6 (170)	18.2 (131)	19.9 (143)	17.2 (124)	
10000–49999	22.2 (188)	18.0 (152)	20.6 174)	20.7 (175)	18.5 (156)	
50000–350000	19.3 (169)	20.5 (179)	21.2 (184)	19.8 (173)	19.3 (168)	
>350000	17.6 (145)	18.3 (151)	19.9 (164)	19.5 (161)	24.6 (203)	0.001

^1^ Values are expressed as mean (95% confidence interval) and proportions (n).

^2^ *p* values were obtained by ANOVA and Pearson chi square for normal continuous and categorical variables, respectively.

^3^ Number of participants according to quintiles of monetary daily diet cost (€/d).

^4^ Number of participants according to quintiles of monetary daily diet cost standardized to 1000kcal (€/1000kcal/d).

^5^ University degree.

^6^ Percentage expressed as proportion within region.

^7^ Percentage expressed as proportion within community size.

Energy intake and energy overreporting increased with monetary daily diet cost, whereas energy underreporting decreased (S2 Table). The opposite trends were observed for monetary daily diet cost per 1000kcal (€/1000kcal/d). Carbohydrate intake decreased with increasing monetary diet cost under both metrics. The opposite was found for protein intake and KIDMED score (S1 Table). Fat intake increased with monetary daily diet cost (€/d) but not with the energy-adjusted metrics.

Table 2 shows the association between adherence to the Mediterranean diet measured by the KIDMED index and monetary daily diet cost calculated as €/d and €/1000kcal/d. Higher adherence to the Mediterranean diet was 0.71€/d and 0.28€/1000kcal/d more expensive than low adherence.

**Table 2:** Association between adherence to the Mediterranean diet, measured by the KIDMED index, and monetary daily diet cost (€/day and €/1000 kcal/day)^1^.

	n	€/day (95% CI)	€/1000 kcal/day (95% CI)
*KIDMED index*^2^			
Low adherence	135	2.64 (2.39; 2.88)	1.34 (1.22; 1.46)
Intermediate adherence	1642	3.03 (2.96; 3.10)	1.53 (1.49; 1.56)
High adherence	1485	3.35 (3.27; 3.42)	1.62 (1.58; 1.66)
P for trend		P<0.001	P<0.001

^1^ Adjusted for sex, age, region, community size, maternal education, and energy over- and underreporting.

^2^ Low adherence: KIDMED index 0 to 3 points; Intermediate adherence: KIDMED index 4 to 7 points; High adherence. KIDMED index more than 7 points.

**Fig 1:**
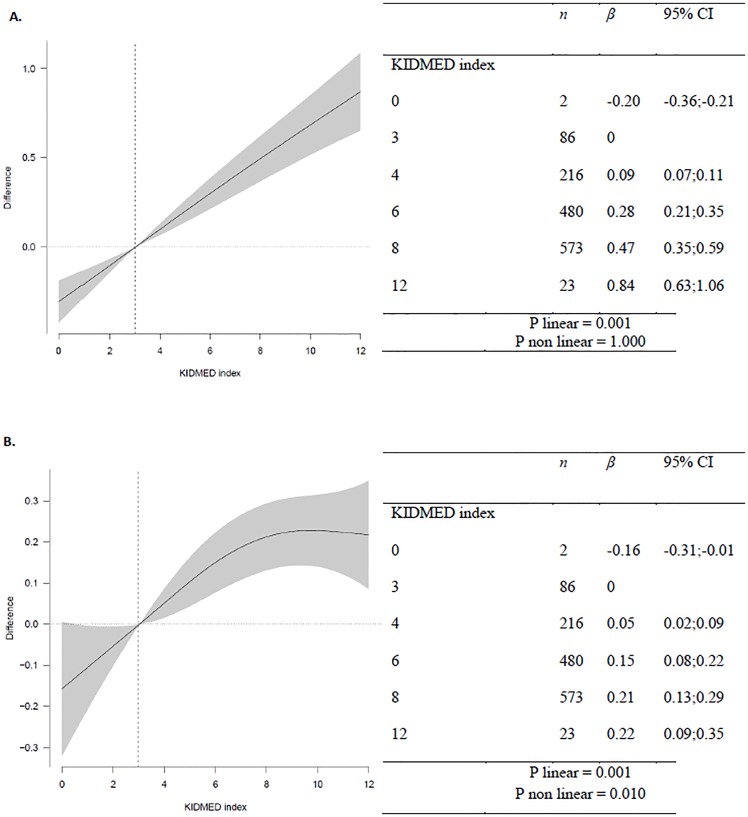
Spline regression of monetary daily diet cost and the KIDMED index. A = €/day; B = €/1000kcal/day. Models were adjusted for sex, age, region, community size, maternal education, and energy over- and underreporting.

Fig 1 shows that the spline curve of the confounder-adjusted association between the KIDMED index and monetary daily diet cost (€/1000kcal/d) reached a plateau beyond a KIDMED score of 8 points, which explains the significant non-linear association. No nonlinear association was observed between monetary daily diet cost (€/d) and the KIDMED index.

**Fig 2:**
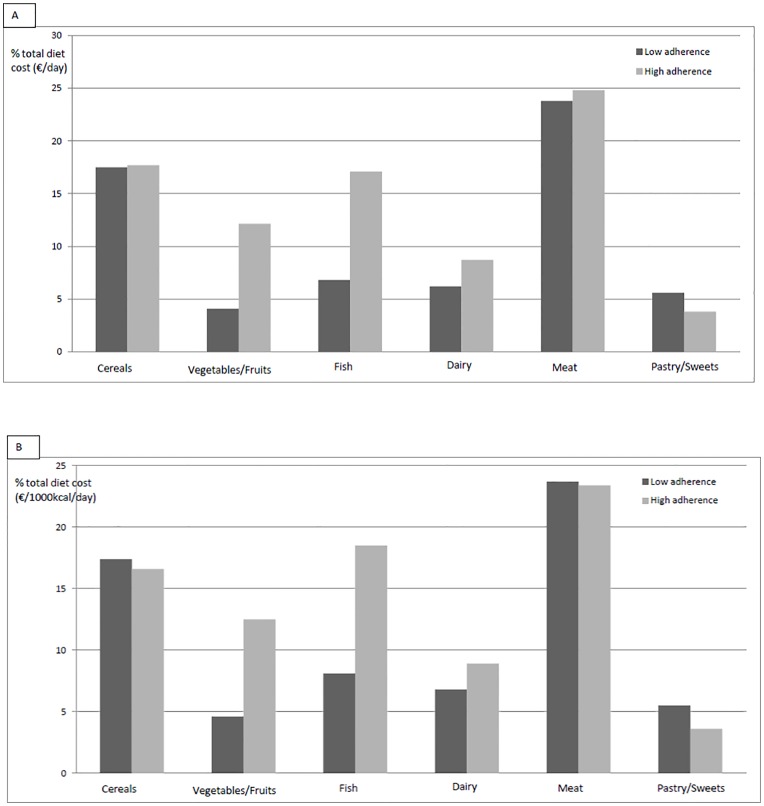
Daily food cost as percentage of total diet cost according to low and high adherence to the KIDMED index. A = €/day; B = €/1000kcal/day adjusted for sex, age, region, community size, maternal education, energy over and underreporting, and Bonferroni adjusted pairwise comparison of means. P<0.05 for all differences, with the exception of meat and cereals. Low adherence = 0 to 3 points on the KIDMED index; high adherence = more than 7 points on the KIDMED index.

Participants with high adherence to the Mediterranean diet spend more money (€/d and €/1000kcal/d) of their food budget for fish, dairy products, and fruits and vegetables compared to their peers with poor adherence (Fig 2B); the opposite was true for pastries and sweets.

**Fig 3:**
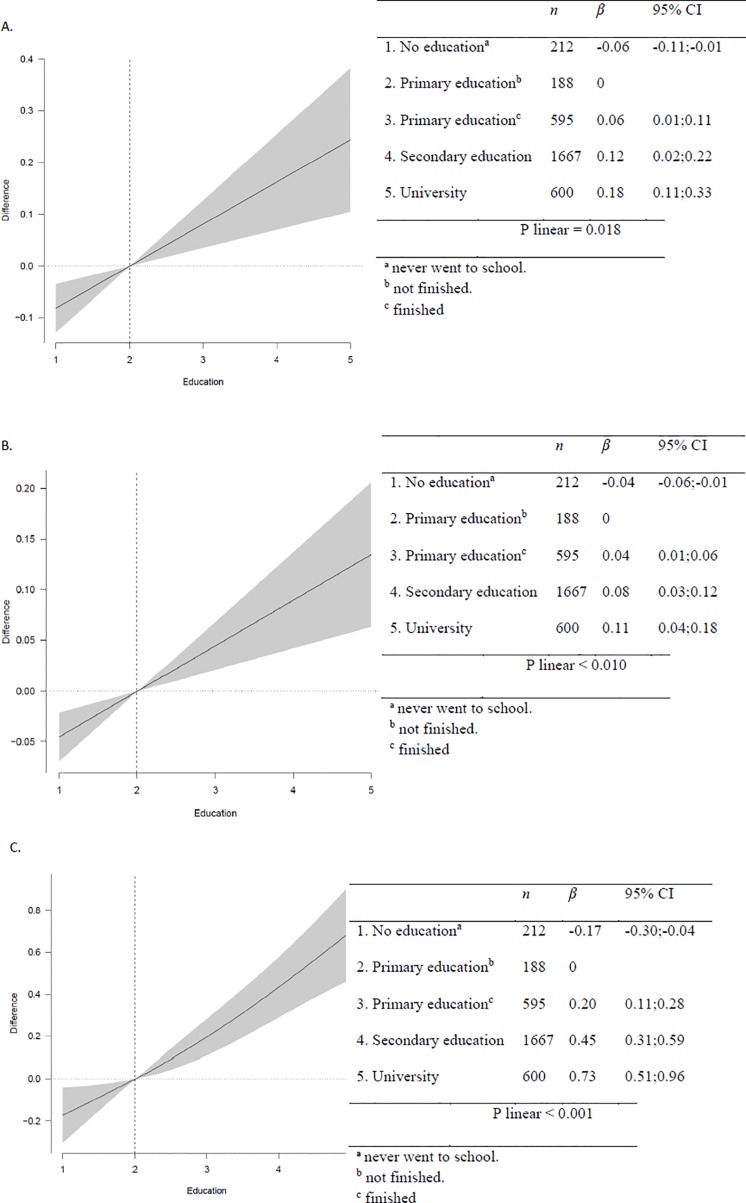
Association of maternal educational status with monetary daily diet cost and diet quality. A = €/day; B = €/1000kcal/day; C = KIDMED index. Models were adjusted for sex, age, region, community size, maternal education, and energy over- and underreporting.

Fig 3 shows the positive association of maternal education with monetary daily diet cost (€/d and €/1000kcal/d) and diet quality measured by the KIDMED index. The diet of the children of highly educated mothers cost 0.24€/d and 0.13€/1000kcal/d more than that of children whose mother never went to school.

Finally, we performed a sensitivity analysis to test if our main finding, the association between monetary diet cost and diet quality, was consistent across age groups and sex. The slope of the regression line was similar (S2 Table).

## Discussion

Higher overall diet quality, measured by adherence to the Mediterranean diet according to the KIDMED index, was more expensive than less healthy choices. The diet was both more expensive and healthier in families with more highly educated mothers. These findings did not differ by type of measurement metric (€/d and €/1000kcal/d).

A recently published meta-analysis including 27 studies conducted in adult populations showed that high-quality diets cost an average of $US 1.54 per 2000 kcal/day more than those with lesser quality [10]. Less is known about the association between monetary diet cost and diet quality in youth. The DONALD study, an open cohort study of German children and adolescents, reported a direct association between monetary daily diet cost (€/d) and diet quality, but only in those participants with high quality scores, defined as exceeding the median of the Nutrient Quality Index (NQI) and the Healthy Nutrition Score for Kids and Youth [26]. A study in Swedish children showed that higher diet quality, determined by the 2005 Healthy Eating Index, resulted in higher monetary daily diet cost (€/1000kcal/d) [11]. The cost differential of 0.34€/1000kcal/d between low and high diet quality found in the Swedish study is comparable with the findings in the present study.

Higher adherence to the Mediterranean diet by Spanish adults is more expensive than the westernized diet [9]. The KIDMED index was developed to determine Mediterranean diet adherence in youth [13]. Higher KIDMED scores have been associated with high nutrient adequacy, a healthier food consumption profile, and lesser weight and abdominal adiposity [14, 19, 20, 27]. In the present study, high adherence to the Mediterranean diet, defined as scoring more than 8 points on the KIDMED index, was significantly more expensive (€/d and €/1000kcal/d) than low or medium adherence. Furthermore, high adherence to the Mediterranean diet was characterized by spending more of the food budget for fruits/vegetables, fish, and dairy (€/d and €/1000kcal/d). Dose-response analysis revealed that scoring more than 8 points did not further increase monetary daily diet cost standardized to a 1000kcal diet. This implies an association between increasing the quality of low- and medium-quality diets and higher monetary diet costs that does not apply to further enhancing high-quality diets. However, this assumption held only if monetary daily diet cost is expressed as € per 1000kcal and day and not as € per day.

It has been shown that the metric of diet cost is important to the interpretation of results, especially for the cost of food and nutrients [28]. For example, using the price-per-kcal metric (i.e., the price of food energy), vegetables and fruits were more expensive than meat and sausages, whereas the opposite was true for the calculation based on the price-per-day metric. Furthermore, a healthy nutrient-based dietary pattern was more expensive than an unhealthy pattern when standardized to a 2000kcal diet but not when a price-per-day metric was used [10]. Indeed, adjusting food cost by energy intake has been questioned, and some have argued that such adjustment leads to erroneous conclusions [29]. However, Rao and colleagues [10] found that dietary patterns were not significantly affected by the metric of price calculation, perhaps due to prior energy adjustment of dietary patterns or a null association between dietary patterns and energy intake, such as in the present study.

Swedish researchers reported higher monetary diet cost and quality with increasing parental SES [11]. This finding is in line with results of the present study, showing a considerable difference in monetary daily diet cost and adherence to the Mediterranean diet between extremes of maternal education level. At first glance, it seems that 0.25€ per day in monetary daily diet cost between more and less favorable SES is not a great difference. However, in a two-child family this difference would total 350€ per year, without counting the parents’ food intake. It has been shown that low income is closely related with low education in Spain [30]. Therefore, it is reasonable to assume that the proportional difference in food expenditure between families with parents of low versus high education levels is greater than in the aforementioned calculation. In 2000, the average price of 100g of fruits, vegetables, and fish was 1.51€, 1.33€, and 6.58€ [18], whereas that of pastries, soft drinks, and sweets was considerably lower. Following dietary recommendations that promote high consumption of low-energy but nutrient-dense foods such as fruits and vegetables and low consumption of nutrient-empty, energy-dense foods will increase diet quality but also the monetary diet cost. This can be a major barrier to opting for healthy food, especially for families with a low SES. Therefore, it is not surprising that increasing monetary daily diet costs (€/d and €/1000kcal/d) were associated with higher SES, accompanied by a substantial increase in diet quality, in the present study.

A limitation of this study is that the EnKid study data are now 15 years old. From 2000 to 2015, most of the foods characterizing the Mediterranean diet showed stronger price increases than foods not considered typical of this recommended diet. Additionally, Spain has experienced one of the highest unemployment rates in Europe since the economic crisis began in 2008, currently has among the highest poverty rates in the region, especially in households with children, and is one of the leading countries in economic inequality in the Organization for Economic Cooperation and Development [31]. Therefore, it is reasonable to assume that the results of this study underestimate the magnitude of the current association between monetary diet cost and healthy eating, especially among families with low SES—those most affected by the economic crisis. A further limitation is the cross-sectional study design, which precludes drawing causal relationships. Furthermore, 24-hour recalls—and particularly a single day’s recall—have inherent limitations in individual dietary assessment, due to daily variations in food intake. Nonetheless, this study has several important strengths, including a nationwide population-based sample and the availability of national food prices for the same time period as participant recruitment.

## Conclusion

In conclusion, healthy eating–characterized by a better adherence to the Mediterranean diet–was associated with higher actual monetary daily diet cost standardized to a 1000kcal diet. Of particular concern is that low diet quality, accompanied by lower diet costs, was found in youth from families with less favorable socioeconomic conditions.

## Supporting Information

S1 Dataset Dataset used in all analyses. (ZIP)

S1 Table Dietary variables and daily monetary diet cost (€/day and €/1000kcal/day)^1^. ^1^ Values are expressed as means (95% confidence interval) and percentage (n). ^2^Polynomial contrasts used to determine p for linear trend were obtained by ANOVA and Pearson chi square for continuous and categorical variables, respectively.
(DOCX)

S2 Table Linear regression analysis between monetary diet cost and the KIDMED index stratified by sex and age group. ^**1** 1^ Adjusted for age, sex, maternal education, region, community size, and energy over- and underreporting.
(DOCX)
