# Supplementary material for: Monetary Diet Cost, Diet Quality, and Parental Socioeconomic Status in Spanish Youth
Source: PLoS One. 2016 Sep 13;11(9):e0161422. doi: 10.1371/journal.pone.0161422 (PMC5021338; doi:10.1371/journal.pone.0161422)
Supplement: S1 Table — 1 Values are expressed as means (95% confidence interval) and percentage (n). 2Polynomial contrasts used to determine p for linear trend were obtained by ANOVA and Pearson chi square for continuous and categorical variables, respectively. (DOCX) [file pone.0161422.s002.docx]

**S1 Table. Dietary variables and daily monetary diet cost (€/d and €/1000kcal/d)^1^**

|  | | 1^st^ quintile  ^3^(n=652) | 2^nd^ quintile  (n=652) | 3^rd^ quintile  (n=653) | 4^th^ quintile  (n=652) | 5^th^ quintile  (n=653) | *P^2^* | |
| --- | --- | --- | --- | --- | --- | --- | --- | --- |
|  | | ^4^(n=653) | (n=652) | (n=653) | (n=652) | (n=652) |  | |
|  |  | |  |  |  |  |  |  |
| Energy intake (kcal) |  | |  |  |  |  |  |  |
| €/d | 1526 (1474;1579) | | 1830 (1778;1882) | 2008 (1956;2060) | 2283 (2231;2335) | 2714 (2661;2766) | <0.001 |  |
| €/1000kcal/d | 2181 (2121;2241) | | 2156 (2096;2216) | 2155 (2095;2215) | 2050 (1990;2110) | 1814 (1753;1874) | <0.001 |  |
| E% carbohydrate |  | |  |  |  |  |  |  |
| €/d | 48.1 (47.4;48.9) | | 46.2 (45.5 (47.0) | 45.5 (44.7;46.2) | 45.0 (44.2;45.7) | 44.2 (43.5;44.9) | <0.001 |  |
| €/1000kcal/d | 46.6 (45.9;47.4) | | 46.5 (45.8;47.3) | 46.3 (45.5;47.0) | 45.2 (44.4;45.9) | 44.5 (43.7;45.2) | <0.001 |  |
| E% protein |  | |  |  |  |  |  |  |
| €/d | 15.7 (15.4;16.0) | | 17.0 (16.6;17.3) | 17.5 (17.2;17.9) | 17.9 (17.6;18.2) | 18.8 (18.4;19.1) | <0.001 |  |
| €/1000kcal/d | 14.6 (14.3;14.9) | | 15.9 (15.6;16.2) | 17.3 (17.0;17.7) | 18.5 (18.2;18.9) | 20.6 (20.2;20.9) | <0.001 |  |
| E% fat |  | |  |  |  |  |  |  |
| €/d | 38.3 (37.7;39.0) | | 39.1 (38.5;39.8) | 39.4 (38.8;40.0) | 39.2 (38.6;39.9) | 38.6 (38.0;39.3) | 0.498 |  |
| €/1000kcal/d | 40.5 (39.8;41.1) | | 39.7 (39.0;40.3) | 38.9 (38.2;39.5) | 38.6 (37.9;39.2) | 37.1 (36.5;37.8) | <0.001 |  |
| **Table continues** |  | |  |  |  |  |  |  |
|  |  | |  |  |  |  |  |  |
|  |  | |  |  |  |  |  |  |
| **Table continued** |  | |  |  |  |  |  |  |
|  |  | |  |  |  |  |  |  |
| KIDMED index |  | |  |  |  |  |  |  |
| €/d | 6.8 (6.6;6.9) | | 7.1 (7.0;7.3) | 7.2 (7.0;7.4) | 7.3 (7.1;7.4) | 7.6 (7.4;7.7) | <0.001 |  |
| €/1000kcal/d | 6.9 (6.7;7.1) | | 7.1 (6.9;7.2) | 7.2 (7.0;7.3) | 7.4 (7.2;7.5) | 7.5 (7.3;7.6) | <0.001 |  |
| Energy overreporting (%) |  | |  |  |  |  |  |  |
| €/d | 0.2 (1) | | 0.3 (2) | 0.8 (5) | 1.5 (10) | 3.9 (25) | <0.001 |  |
| €/1000kcal/d | 2.1 (14) | | 1.8 (12) | 0.9 (6) | 1.4 (9) | 0.3 (2) | <0.001 |  |
| Energy underreporting (%) |  | |  |  |  |  |  |  |
| €/d | 31.1 (204) | | 17.6 (115) | 15.6 (101) | 10.8 (70) | 8.8 (57) | <0.001 |  |
| €/1000kcal/d | 12.3 (80) | | 11.5 (75) | 15.3 (100) | 16.9 (110) | 28.2 (182) | <0.001 |  |

^1^ Values are expressed as means (95% confidence interval) and percentage (n).

^2^ Polynomial contrasts were used to determine p for linear trend were obtained by ANOVA and Pearson chi square for continuous and categorical variables, respectively.
