# Supplementary material for: Monetary Diet Cost, Diet Quality, and Parental Socioeconomic Status in Spanish Youth
Source: PLoS One. 2016 Sep 13;11(9):e0161422. doi: 10.1371/journal.pone.0161422 (PMC5021338; doi:10.1371/journal.pone.0161422)
Supplement: S2 Table — 1 1 Adjusted for age, sex, maternal education, region, community size, and energy over- and underreporting. (DOCX) [file pone.0161422.s003.docx]

**S2 Table. Linear regression analysis between monetary diet cost and the KIDMED index stratified by sex**

**and age group.^1^**

|  |  | Monetary diet cost (€/d) | | | Monetary diet cost (€/1000kcal/d) | | |
| --- | --- | --- | --- | --- | --- | --- | --- |
|  | n | *β* coefficient | 95% CI | *p* | *β* coefficient | 95% CI | *p* |
| KIDMED index |  |  |  |  |  |  |  |
| *Sex* |  |  |  |  |  |  |  |
| -Boys | 1509 | 0.126 | 0.089;0.164 | <0.001 | 0.037 | 0.022;0.053 | <0.001 |
| -Girls | 1753 | 0.079 | 0.049;0.108 | <0.001 | 0.026 | 0.008;0.046 | 0.003 |
| *Age group* |  |  |  |  |  |  |  |
| -Pre-schoolers | 373 | 0.066 | 0.004;0.128 | 0.037 | 0.026 | -0.013;0.006 | 0.195 |
| -Children | 586 | 0.075 | 0.026;0.125 | 0.003 | 0.020 | -0.003;0.043 | 0.083 |
| -Adolescents | 937 | 0.132 | 0.084;0.181 | <0.001 | 0.034 | 0.013;0.055 | 0.001 |
| -Young adults | 1367 | 0.105 | 0.019;0.142 | <0.001 | 0.032 | 0.013;0.051 | 0.001 |

^1^ Adjusted for age, gender, maternal education, region, community size, and energy over- and underreporting.
